# Legodroid: A Type-Driven Library for Android and LEGO Mindstorms Interoperability †

**DOI:** 10.3390/s20071926

**Published:** 2020-03-30

**Authors:** Alvise Spanò, Agostino Cortesi

**Affiliations:** Dipartimento di Scienze Ambientali, Informatica e Statistica, Università Ca’ Foscari Venezia, via Torino 155, 30170 Mestre-Venezia, Italy; cortesi@unive.it

**Keywords:** Android, LEGO mindstorms, EV3, type-driven development, design patterns

## Abstract

LEGO Mindstorms robots are widely used as educational tools to acquire skills in programming complex systems involving the interaction of sensors and actuators, and they offer a flexible and modular workbench to design and evaluate user–machine interaction prototypes in the robotic area. However, there is still a lack of support to interoperability features and the need of high-level tools to program the interaction of a robot with other devices. In this paper, we introduce Legodroid, a new Java library enabling cross-programming LEGO Mindstorms robots through Android smartphones that exploits their combined computational and sensorial capabilities in a seamless way. The library provides a number of type-driven coding patterns for interacting with sensors and motors. In this way, the robustness of the software managing robot’s sensors dramatically improves.

## 1. Introduction

The LEGO Mindstorms environment represents a natural step forward in the compositional approach to problem solving provided by Duplo and plain LEGO during childhood, by offering an ideal workbench for learning the necessary programming skills for operating with sensors in human–robot interaction and in the development of systems for the Internet of Things (IoT). Unfortunately, there is still a lack of support for interoperability features, such as the interaction of the robot with other non-LEGO devices. This dramatically limits the integration of LEGO Mindstorm robots into more complex systems.

In order to reduce this gap, this paper (which extends [1]) introduces Legodroid, a new Java library for Android for cross-programming the EV3 brick from an Android device through an API relying on type-driven programming principles. Legodroid is freely available at [2].

Our library supports IoT development by allowing full control of sensors and motors on the remote robot while enforcing a methodology that induces the programmer to write robust and sound programs. IoT programming traditionally employs mainstream languages and frameworks, aiming more at achieving technology than improving development methodologies or employing sophisticated programming paradigms. In a field where the integration of multiple devices equipped with sensors and actuators is crucial, manipulating loosely-typed data and employing low-level programming patterns may introduce errors and bugs due to the lack of static validation of code. We believe that imposing a methodology based on a strict type discipline represents a good trade-off between development complexity and benefits in terms of code correctness, improving the overall quality of software only by means of a wise use of the type system.

Legodroid provides a collection of type-driven methodologies designed to train junior as well as senior programmers in the development and production of stable, maintainable, and scalable applications and systems. Interaction with the EV3 brick—the robot CPU—is seamless and it allows the programmer to focus on the program architecture and algorithms, rather than on communication and related mechanisms. The robot system is programmed by means of one big callback—we call it the lego main.

The main novelties of Legodroid with respect to state-of-the-art tools enabling the interaction with LEGO Mindistorm devices can be summarized as follows:

- Legodroid unleashes the computational power of an external device hosting and running a program that communicates with the robot through its ABI. The application business logic entirely runs on the Android side. The interaction with the brick is seamless and it allows the programmer to focus on the program architecture and algorithms, rather than on communication and related mechanisms. The robot is programmed by means of one big callback—we call it the *lego main*. This is the only entry-point for interacting with sensors and motors connected to the brick. The benefits of this approach include:
  - *computing power*: Android devices ranging from mobile phones to tablets are equipped with a much more powerful CPU than the EV3, enabling time-consuming algorithms to run on the mobile side;
  - *sensors*: robot’s sensors can be managed by procedures written in a higher level language. Moreover, a number of devices such as cameras and microphones can be exploited by the programmer for adding additional eyes and ears to the LEGO robot, processing data coming from these sensors as well;
  - *development environment*: Android Studio [3] is a powerful IDE with debuggers, code analyzers, and other tools aiding developers in writing apps;
  - *third party technologies*: the entire Android SDK lies at the programmer’s fingertips, including its versatile UI/UX subsystem, reusable services performing with a number of common system-wide tasks, plus a great variety of third party libraries that are available for Android and are suitable for inter-operation with LEGO Mindstorms—e.g., OpenCV for Android [4] for bringing image recognition to the robot through the smartphone camera.
- Type-driven development relies on an accurate type design, code reuse, and polymorphism, requiring validation when compiling the code rather than writing algorithms with untyped or barely typed data. The basic idea is that “a strong type system can not only prevent errors, but also guide you and provide feedback in your design process” [5]. Type-driven programming brings the type-safe coding discipline coming from the world of functional languages to the world of IoT programming and mainstream application development.

The paper is structured as follows. In Section 2, we discuss related work. Section 3 outlines the methodological principles that characterize our proposal. Section 4 delves into the breakdown of the modules the library consists in, presenting UML diagrams, code excerpts, and samples for detailing the API. Section 5 formalizes the type-driven programming patterns. Section 6 explores advanced aspects and uses of the library, such as improving the quality of data read from sensors or dealing with concurrent access to the EV3 brick. Section 7 contains an in-depth impact evaluation as well as a usability evaluation of the library. Section 8 provides the conclusions.

## 2. Related Work

LEGO Mindstorms is an educational platform supporting a number of SDKs for programming the main control unit, namely the EV3 brick. There are mainly four ways to program the robot:

1. RoboLab [6] is an educational language for workflow-oriented visual programming in the likes of Scratch [7], officially distributed by LEGO and meant for kids to learn coding and problem solving.
2. leJOS [8] is a Java porting of the LEGO NXT Kit [9], providing classes and methods for moving EV3 motors and reading sensors with a object-oriented API; programs run directly on the EV3 device.
3. Flashing the brick ROM with a custom firmware is also an option, arguably addressed to those willing to take over the system and reprogram it from scratch.
4. An application can connect to the EV3 brick through a TCP socket and start sending commands, i.e., structured streams of bytes, formatted according to the EV3 ABI specification defined in the *EV3 Communication Developer Kit* [10] On the EV3 side, a server process is constantly up and listening to incoming WiFi (At the time of writing, Legodroid does not support WiFi connections, as the Bluetooth counterpart is preferable in most cases. A WifiConnection class is expected by design though and will be added in a future update.) or Bluetooth connections, serving clients by processing incoming commands and sending replies, as documented in the *EV3 Firmware Developer Kit* [11]

Option 1 strongly suffers by the limitations of the RoboLab toy-language. Option 2 is limited by the poor computational power of the EV3 brick, as it is based on programs just running on it (The EV3 CPU is a 300 MHz TI Sitara AM1808 (ARM926EJ-S Core) with 64 MB of RAM.). Option 3 is not satisfactory either, as it introduces unnecessary complexity: it requires the complete rewriting of all system-level interaction and communication components. Option 4 is the one adopted by our proposal: we delve now into the comparison with the most relevant competitors.

### 2.1. leJOS and NXT

We first compare our library with the most widespread toolkit for programming LEGO Mindstorms, leJOS. Up to version 0.9, the leJOS library is based on the original LEGO Mindstorms NXT system [12]. Therefore, the discussion on leJOS applies also to NXT. The major difference between leJOS and our proposal is that Legodroid is a Java library for Android, thus programmers will design programs that run on the smartphone instead of directly on the EV3 CPU. This enables the development of more sophisticated applications and algorithms, since the full Android library machinery can be exploited, as already discussed in Section 1.

The leJoS version 0.9 supports Android cross-programming as a separate library module, called leJOS PC API [13], making this an ideal candidate for comparisons with Legodroid. The level of abstraction provided for accessing remote sensors and motors by its APIs is similar to Legodroid: sensors are represented by objects providing methods for reading input values in basic datatype formats, such as integers, floats, and arrays. Legodroid offers an additional feature: data coming from sensors is pre-processed through a spooler thread dispatching each incoming value to the thread that originally performed the reading operation, wrapping results within Future computations. This allows programmers to exploit concurrency in a transparent way, whereas leJOS just delivers lower level primitives for controlling the EV3. Therefore, leJOS programmers need to put extra care when dealing with Android activities and components. For instance, heavy usage of Handler and related asynchronous patterns is required for exchanging incoming sensor data with the UI or other active threads [14].

Moreover, Legodroid features type-driven patterns for accessing the EV3, its sensors, and motors, and supports the extension of the API in a disciplined way thanks to a multi-layer design, as discussed in Section 4.1 and Section 5: such features have no correspondence in leJOS. On the downside, currently Legodroid does not support programs natively running on the EV3 brick, as it only supports cross-programming through Android.

### 2.2. LPCCA

Among the few libraries for controlling LEGO Mindstorms from an Android remote device, the most significant is LPCCA [15]. It consists of a leJOS PC API wrapper running as a service on the Android side and controlling the EV3 remotely via a Bluetooth channel. This service-based design allows for multiple interfaces: a web interface as well as a native Android UI are equally feasible. The API is similar to other wrappers for controlling the EV3, providing Java interfaces and classes for moving motors and reading sensors. Compared to Legodroid, LPCCA does not support code robustness and sound programming practices. Instead, for sophisticated typing techniques, LPCCA stakes everything on technological features such as a visual configurator of the EV3 I/O ports, support for USB connections, compatibility with web-based applications, and low latency communication. From a programming point of view, LPCCA as well as leJOS suffer from several limitations, such as the presence of singletons in the implementation, making the library not able to scale with multiple EV3 or unsuitable for multi-threaded Android apps involving concurrent access to sensors.

### 2.3. Other Solutions

More recent contributions [16,17] face the problem of programming a robot from a remote Android device, focusing more on applicative and technological aspects rather than providing APIs for controlling the robot in a sound way.

### 2.4. LEGO Mindstorms as an Educational Environment for Learning Sensor Programming

Using LEGO Mindstorms for teaching programming skills for embedded systems design has already been explored in literature [18,19,20]. In particular, several educational experiments have been discussed [21,22,23], reporting positive feedback of LEGO Mindstorms as a development platform by college students [24]. In recent years, remote controlling of LEGO Mindstorms robots from Android devices has been explored as well [15,17], also as a platform for learning coding [16]. These works, though, do not put the emphasis on disciplined coding practices such as accessing sensors in a type-sound way: they rather focus on technological issues, such as communication between the mobile device and the robot and on the interaction with sensors and actuators. Compared to such works, our proposal targets a more experienced audience, assuming high level coding skills and a certain level of education on programming languages. These papers agree on the low computational power of the EV3 CPU as the main motivation for the need of hosting computations on a remote device, since the manipulation of data coming from the sensors fully performed by the EV3 CPU lacks efficiency.

## 3. Methodological Principles

We adopt the type-driven approach to software development. This approach has increased its popularity in the academic and professional communities over the last decade: the Haskell and F# communities have been promoting the benefits of designing with types [25], showing how writing programs adopt the Hindley–Milner type system [26] improves the programmer’s understanding of the static and dynamic properties of software [27]. In addition, more advanced languages based on dependent type systems [28] such as Idris [29] brought type-driven development to a form of *assisted* programming guided by rich type information.

The main methodological programming principles that Legodroid aims to enforce can be summarized as follows:

(i) *Use higher-order functions* [30]. Fine-grained custom behaviors can be formulated via lambda expressions, which shift the focus on parametric polymorphism rather than subtyping. The higher-order function approach has been adopted by mainstream languages in the recent years and is nowadays accepted by the OOP community as a right way for customizing the behavior of a generic function [31]
(ii) *Never allow the programmer declare uninitialized variables and force her to construct objects in a valid state*. Nullness checking is crucial: adding Java annotations @NotNull and @Nullable, combined with an aggressive use of the final qualifier, raises the code quality in a sensible way [32]. This has an impact on how classes and constructors are designed. Avoiding no-argument constructors discourages creating empty uninitialized objects that eventually have to be populated by calling setters. This in turn discourages unneeded mutable data [33], thus reducing the overall statefulness of a program, which is responsible for runtime errors due to state invalidity [34].
(iii) *Reduce side effects to the minimum*. Mutable data structures in most imperative programs happen to be involuntary, since mutability is the default condition for variables and fields in most mainstream languages. Overuse of assignment is a common source of bugs, especially when concurrent code is involved, whereas immutable data tend to lift errors up to the type level [35]. Manipulating immutable data types does not make code execution slower, since most modern languages rely on call-by-reference argument passing and data are never copied unless explicitly [36].
(iv) *Use strong types even for intermediate results*. Languages with extensible records and variants [37] allow for an accurate representation of the results of temporary computations. In Java, an advanced use of types and generics [38] can literally guide the programmer to the correct implementation. Each computational step is represented by a strong type. Any invalid sequence of operations would be rejected by the compiler. Control flow becomes data flow; and data are ultimately validated by type checking [39].

## 4. Architecture of the Library

**Figure 1:**
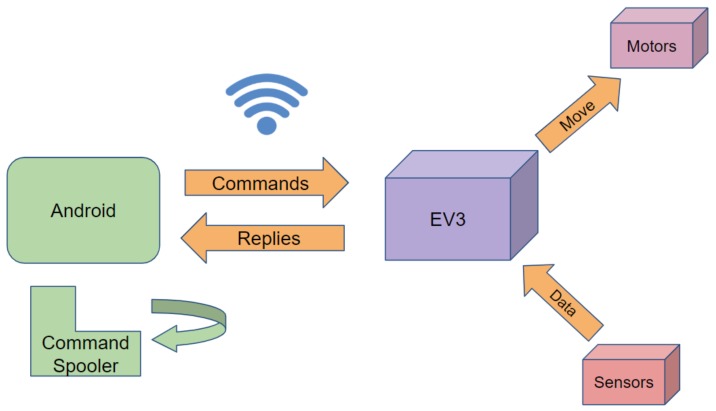
Components of an Android + LEGO Mindstorms system using Legodroid.

In this section, we describe the architecture of the Legodroid library as well as the functionalities and the features provided by the main packages. Figure 1 depicts a high level diagram representing the components of a Legodroid-based Android app interacting with the EV3 and its sensors and motors. The *command spooler* consists in a stealth service running as a thread in the app, constantly processing outgoing commands and incoming replies to/from the EV3.

### 4.1. Package Structure

As far as the code architecture is concerned, the library is designed with three layers of API. Each layer strictly wraps the underlying one and supports extensions.

**Low level API**. It deals with serialization and byte-level manipulation of commands for communicating with the EV3 brick according to the *EV3 Communication Developer Kit* specification. The comm sub-package, detailed in Section 4.3, contains the Bytecode class, aimed at building commands by appending op-codes and manipulating parameters at the byte level in a straightforward way. Users willing to extend the library with new commands can limit use of such low-level primitives to small self-contained methods.
**Mid level API**. Class Api (For the sake of brevity, we may refer to the EV3.Api nested static class as Api) provides the core primitives for interacting with EV3, such as reading SI or PCT values from a sensor. The *EV3 Firmware Development Kit* defines these as half-baked data types translating, respectively, into float and short in Java. Extending the library at this level means to add new methods implementing EV3 instructions that are currently unsupported by Legodroid, manipulating arrays of floats or short according to the specification in Section 4 of the *EV3 Firmware Developer Kit*.
**High level API**. The Api class offers a family of getter methods constructing strong-typed handles to sensors and motors defined in the plugs package. Such handles exhibit methods performing high-level operations over sensors and motors and are distinct classes within the plugs sub-package. Extending the library at this level means to extend the Api class with new methods constructing new handles, which provide the methods implementing new commands for the brick in the same way as classes in plugs do.

Notice that the Api class offers two among the three layers of API mentioned above: public methods form the high level API, whereas the mid level layer consists of protected methods. From a user perspective, the high level methods are enough in most situations, allowing operations over sensors and motors in an straightforward way. Mid-level methods of getSiValue(), getPercentValue(), and execAsync() are not enough, in number, to justify the architectural overhead of an additional class. Users willing to implement new high-level methods have all they need at their fingertips.

In the next subsections, we introduce the main packages of the library. When dealing with function types, the arrow notation is syntactically more convenient than functional interface names. Assuming that ⌀ represents the unit type, the following notations hold:

$$\begin{array}{ccc} \mathrm{Function}<A,\;B> & \equiv & A\to B\\ \mathrm{Consumer}<T> & \equiv & T\to ⌀\\ \mathrm{Runnable} & \equiv & ⌀\to ⌀ \end{array}$$

### 4.2. The Root Package: legodroid.lib

**Figure 2:**
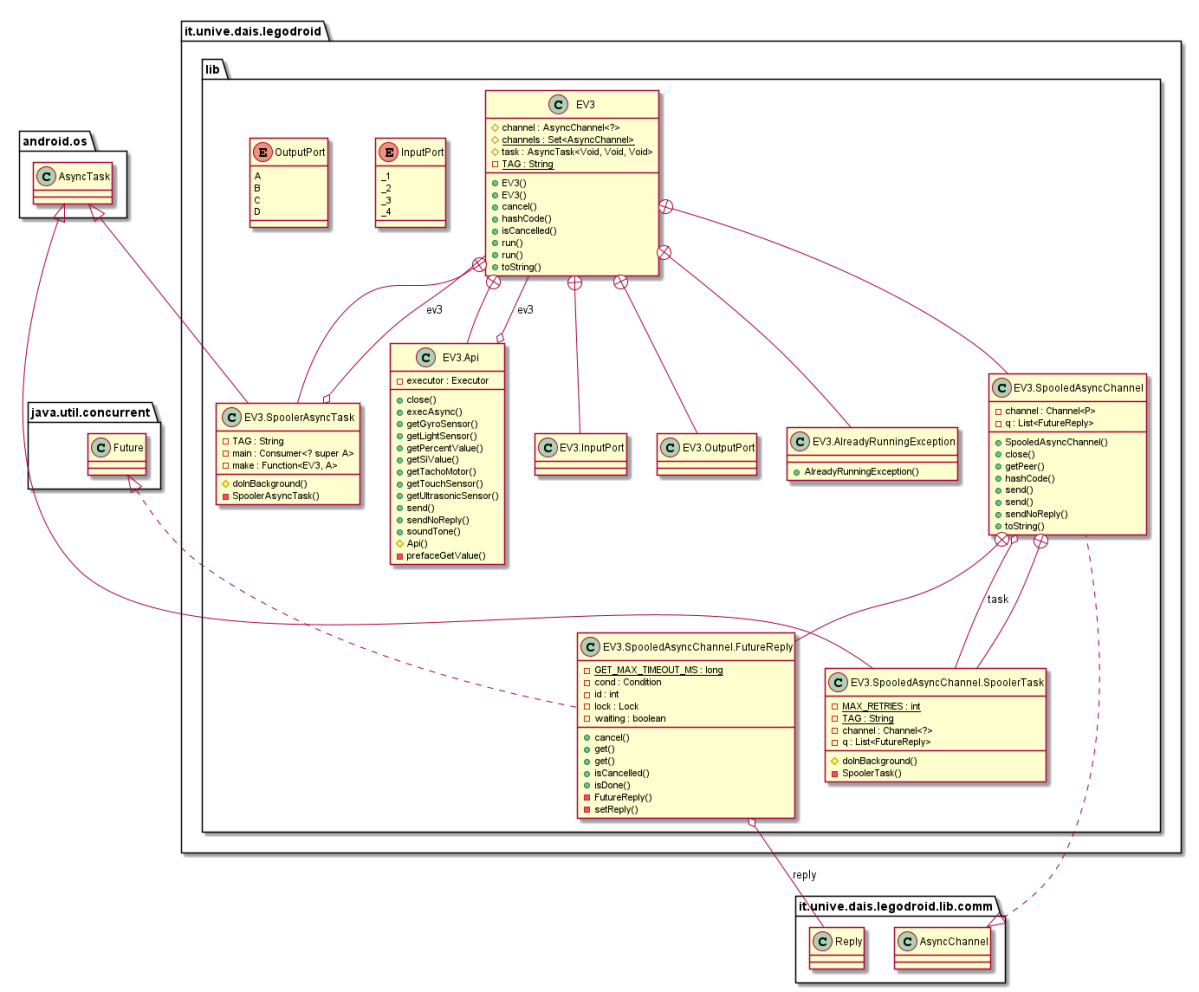
UML Class Diagram of package legodroid.lib.

The root package contains the main classes for programming with Legodroid, as shown in Figure 2. Class EV3 lies at its core and exhibits most of the type-driven practices. An instance of type EV3 represents a physical instance of the EV3 brick. It executes a callback as the main function for that brick. It runs on the Android device as a standalone thread which constantly communicates with the brick in a transparent way.

The run() method picks an argument of type Consumer<Api>, i.e., a function Api→⌀, and executes it in another thread, trapping and logging any unexpected exception. It also guarantees that only one callback is running at any given time on the brick. Class EV3 does not provide any method for interacting with sensors and motors: these functionalities are provided by the Api class. An object of type Api is passed to the Consumer<Api> callback provided by the programmer: there is no other way of obtaining an object of type Api. Notice that the EV3 sensors and motors cannot be accessed from outside of the lego main: this prevents error-prone coding habits such as dealing with objects in an invalid state. See Pattern 2 in Section 6 for further details.

### 4.3. The Communication Package: legodroid.lib.comm

**Figure 3:**
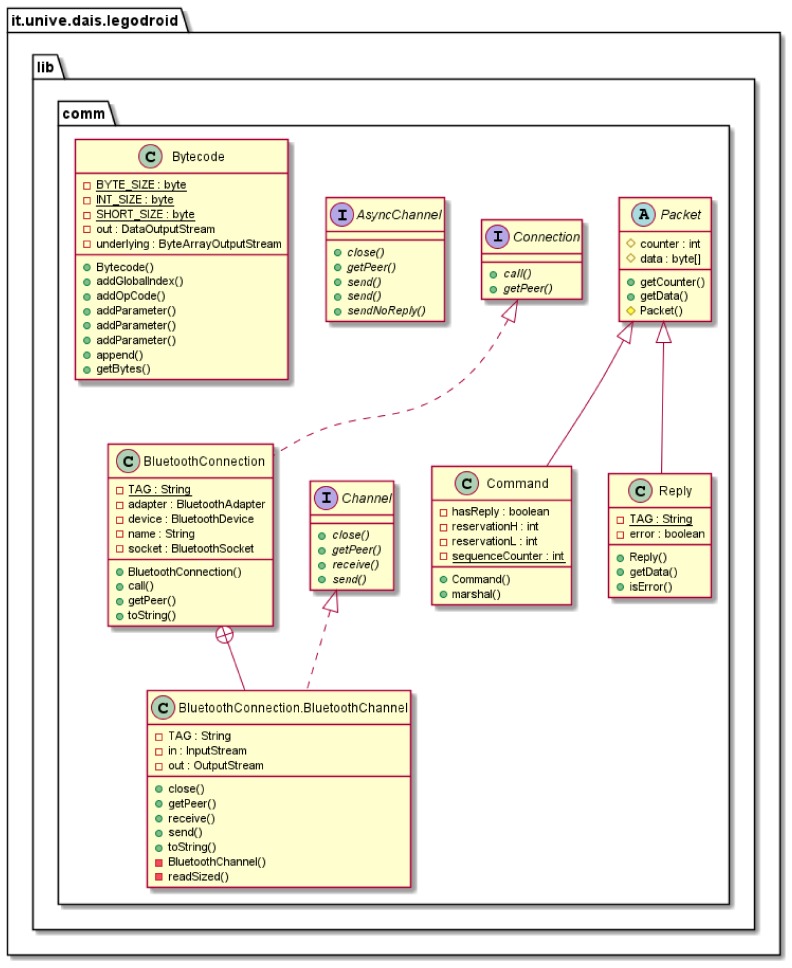
UML Class Diagram of package legodroid.lib.comm.

Package legodroid.lib.comm depicted in Figure 3 provides the communication facilities. Channels represent the basic abstractions offering communication primitives: a Channel can *send* a Command and *receive* a Reply, both of which are subclasses of Packet. Low-level communication with EV3 is based on exchanging data as untyped byte arrays formatted according to the official specification defined by LEGO in the *EV3 Communication Development Kit*: *direct commands* sent by the client and consequent replies coming from EV3 require a byte-per-byte encoding, which includes a header followed by a an extra sequence of bytes carrying the custom content of each request; the header consists of fixed byte fields such as the length of the packet, the sequence number, the command type, the attached data, etc. Class Const binds all C-style preprocessor symbols defined in the official header files as static numeric constant fields in Java, mostly used by the Bytecode class for serializing commands.

The Connection<P, C> interface represents the contract for constructing channels of type C, where C⪯Channel<P> and the type parameter P represents the peer. A Connection<P, C> is also a subtype (We denote the subtype relation as T⪯S, where T and S are types) of the Callable<C> interface:

Connection<P, C⪯Channel<P>>⪯Callable<C>≡⌀→CΔIOException

The functional approach is nowadays considered more versatile than the classic factory pattern  [40] and fits better with the library type-driven principles.

It is worth noting that Connection<P, C> is not a functional interface, as it includes an additional method getPeer() for retrieving the peer of type P. Being a subtype of Callable, however, makes it compatible with a functional interface, enabling advanced manipulation through higher order functions, such as: 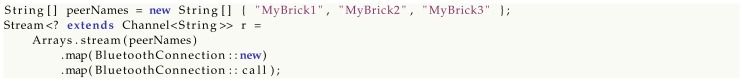


The generic P is unconstrained and unrelated to the return type of the Callable; e.g., in class BluetoothChannel, it is bound to String as the argument type of its constructor.

### 4.4. The Sensors and Motors Package: legodroid.lib.plugs

**Figure 4:**
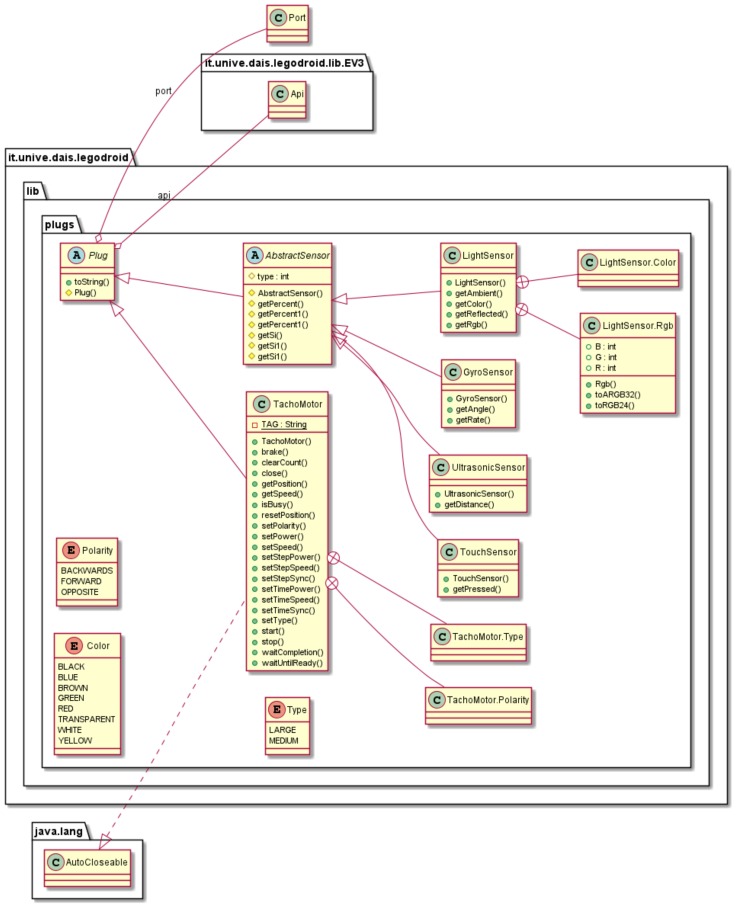
UML Class Diagram of package legodroid.lib.plugs.

Figure 4 depicts the classes representing sensors and motors contained in package legodroid.lib.plugs. Classes like GyroSensor, LightSensor, and TouchSensor provide methods for reading sensors and moving motors in a typed way, where:

1. ports are distinct enum types EV3.OutputPort and EV3.InputPort;
2. minor flags representing motor polarity and type (Refer to Section 4.9 of the *EV3 Firmware Development Kit*, op-codes opOutput_Polarity and opOutput_Set_Type) are enum types as well;
3. all sensor and motor classes inherit from a common superclass Plug<P>, where P represents the port type: this makes subclasses instantiate the generic P with some concrete type at inheritance time;
4. sensors inherit from a common abstract class AbstractSensor: protected methods getPercent(), getPercent1(), getSi(), and getSi1() are commodities for quickly implementing actual sensor subclasses.

Classes included in this package behave as handles for accessing LEGO accessories, such as sensors and motors, connected to the I/O ports. As explained in Section 5, extending the library for supporting new accessories requires just to extend the abstract class Plug or AbstractSensor and to implement the relevant communication primitives on top of the Mid level API.

### 4.5. The Utilities Package: legodroid.lib.util

**Figure 5:**
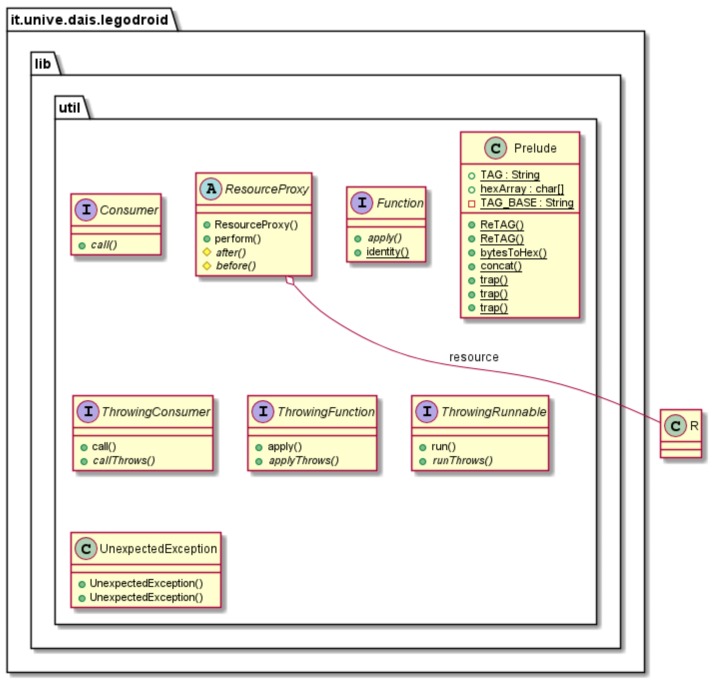
UML Class Diagram of package legodroid.lib.util.

Figure 5 depicts the UML class diagram of the legodroid.lib.util package, offering general utilities in the form of static methods in class Prelude as well as the definition of functional interfaces [41] for supporting older versions of Android preceding Java 8. Functional interfaces are unavailable in the Android SDK prior to version 24, whereas Legodroid targets version 21: interfaces Function and Consumer reproduce the respective functional interfaces.

Specialized functional interfaces supporting exceptions are defined as well: ThrowingConsumer<T, E> extends Consumer<T> adding an extra type parameter E⪯Throwable that statically tracks the exception thrown by the call() method. Java method signatures with throws declarations can be represented by a special arrow notation including the exception information:

$$\begin{array}{ccc} \mathrm{ThrowingFunction}<A,\;B,\;E⪯\mathrm{Throwable}> & \equiv & A\to B\Delta E\\ \mathrm{ThrowingConsumer}<T,\;E⪯\mathrm{Throwable}> & \equiv & T\to ⌀\Delta E\\ \mathrm{ThrowingRunnable}<E⪯\mathrm{Throwable}> & \equiv & ⌀\to ⌀\Delta E \end{array}$$

In order to make such interface subtypes of the respective functional interfaces, an extra method callThrows() adds the throws E declaration, whereas the call() method inherited by the parent functional interface is overridden with the following default semantics: it invokes method callThrows() and traps any exception, converting it from type E to an unchecked RuntimeException.

Class Prelude is a container for utility functions, among which trap() is arguably the most useful: it picks a ThrowingFunction<T, E>, and an argument of type T, applying the former to the latter within a try-catch block trapping any exception. Overloaded implementations of trap() are provided for every exception-throwing functional interface:

$$\begin{array}{cccc} \mathrm{trap} & : & \forall \alpha \;\beta \;(\gamma ⪯\mathrm{Throwable}).\;(\alpha \to \beta \Delta \gamma )*\alpha \to \beta \Delta ⌀ & \left(\mathrm{ThrowingFunction}\right)\\ \mathrm{trap} & : & \forall \alpha \;(\beta ⪯\mathrm{Throwable}).\;(\alpha \to ⌀\Delta \beta )*\alpha \to ⌀\Delta ⌀ & \left(\mathrm{ThrowingConsumer}\right)\\ \mathrm{trap} & : & \forall \alpha ⪯\mathrm{Throwable}.\;(⌀\to ⌀\Delta \alpha )\to ⌀\Delta ⌀ & \left(\mathrm{ThrowingRunnable}\right) \end{array}$$

Finally, class ResourceProxy generalizes a resource acquisition type-driven pattern and will be described in detail in Section 5.

## 5. Type-Driven Patterns

In this section, we introduce the most interesting type-driven programming patterns used in Legodroid. Such patterns lead the programmer to gain access to EV3 sensors and motors in a disciplined way:

- in order to access EV3 motors and sensors, the programmer needs an object of type Api, which can only be obtained as argument passed to the callback of type Consumer<Api> that must be provided to the EV3.run() method;
- an object of type EV3 is therefore needed, which represents a physical EV3 brick connected to the Android device and its constructor requires an object of type Channel;
- a Channel can be created only by means of an object of type Connection: e.g., class BluetoothConnection implements Connection<String, BluetoothChannel>, where the String type argument represents the peer and BluetoothChannel is the return type;
- a Connection requires the peer at construction time: e.g., BluetoothConnection requires a string with the EV3 brick name in order for Bluetooth pairing to take place.

In Java, this sequence of operations and requirements translates into the following pipeline: 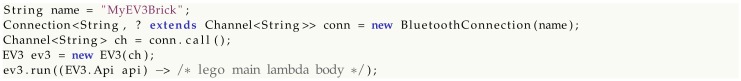


Notice that binding each step to a variable requires a wise use of Java generics and wildcard. This is an equivalent but more compact representation: 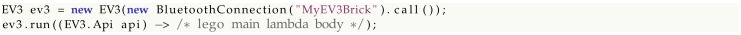


Observe that in the last code snippet there is no room for runtime errors: the pipeline is strictly governed by types, in such a way that any mistake a human programmer could possibly inject into it would lead to a compile time error.

**Figure 6:**
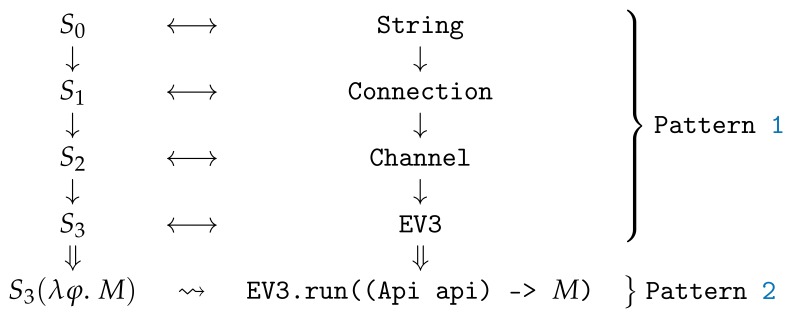
Pipeline of Pattern 1 and Pattern 2.

The diagram depicted in Figure 6 clarifies such type-driven pipeline, where each state $S_{i}$ on the left side corresponds to a specific type on the right side. Two distinct patterns are at work here: Pattern 1 deals with state transformations, whereas Pattern 2 appears on the last line, where the *lego main* callback is involved.

**Pattern** **1** (Type as Evidence)**.** *In order to ensure that a given set of operations φ becomes available only after some state $S_{k}$ (of type $S_{k}$) has been reached within a sequence of increasingly mutating states $S_{1}\;\ldots \;S_{n}$ such that 1≤k≤n and n>0, the following pattern can be followed:*

- the set of operations $\varphi =\{f_{1}\left(\right)\;\ldots \;f_{n}\left(\right)\}$ can be translated into methods of a stateless object of type A;
- *each state $S_{i}$ can be translated into an object of type $S_{i}$ for i∈[1,n]:*
  - each object $S_{i}$ holds the information for the state $S_{i}$;
  - an object of type $S_{i}$, for i>1, can only be constructed by providing an argument of type $S_{i-1}$, i.e., the previous state;
  - the initial state $S_{0}$, implemented by an object of type $S_{0}$, must be constructed explicitly from scratch;
- objects of type A can only be constructed given an argument of type $S_{k}$.

In Legodroid, the stateless type A mentioned in Pattern 1 is class Api. Notably, types are states and constructors act like functions from states to states, where the constructor for type $S_{i}$ is a function $S_{i-1}\to S_{i}$. This pattern works under the assumption that the only way to produce a state $S_{i}$ is by constructing its type as a function of the previous state $S_{i-1}$.

In the last stage of the pipeline depicted in Figure 6, Pattern 2 is at work: consider $S_{3}$ as a special functional state supporting application, applying it to a lambda argument whose parameter is the set of operations $\varphi =\{f_{1}\left(\right)\;\ldots \;f_{n}\left(\right)\}$ and whose body is the lego main code block *M*. This translates into the invocation of the EV3.run() method with a callback argument that is parametric over an Api object.

**Pattern** **2** (Limiting Resource Access)**.** *Users willing to access a resource R must provide a callback f by either defining a lambda, an anonymous class or a functional object (In Java 8+, all the mentioned language constructs are equivalent type-wise) parametric over the resource R. The owner applies R to f and can control what happens before and after the function application.*

Pattern 2 has a number of applications, as it allows in general to perform some task before and after executing the given callback, for example:

- synchronizing access to a resource by locking and unlocking a mutex;
- trapping exceptions by surrounding a function call with a try-catch block (Method Prelude.trap() in package legodroid.lib.util is an example of this usage of Pattern 2).

This behavior can be described as a functional resource proxy and is offered by Legodroid as a generic class included in the it.unive.dais.legodroid.lib.util package: 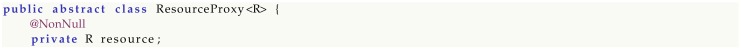 
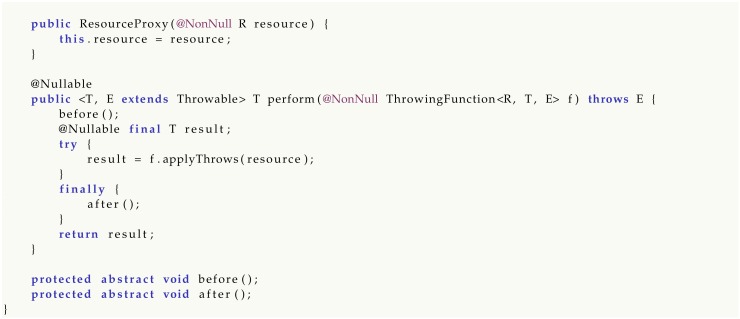


By inheriting ResourceProxy and overriding methods before() and after(), programmers can customize the behavior of what to do before and after the access to the resource of type R, which is limited to the scope of the ThrowingFunction callback. Notice that this pattern does not prevent the user from saving the pointer to the resource for later use outside of its controlled scope: 
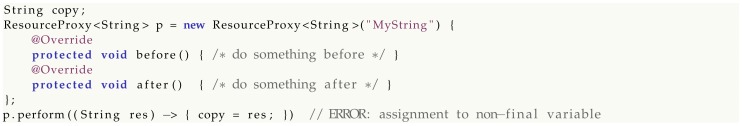


The Java compiler, however, forbids assignments to a non-final variable defined outside of a closure, rejecting such scenarios.

## 6. Advanced Features

In this section, we discuss some advanced aspects of programming with Legodroid.

### 6.1. Reusing EV3

Reuse of the EV3 is intended in two ways:

1. Once a lego main callback returns, another callback can be run—there can be up to one running lego main at any given time on the same EV3 object;
2. the same EV3 object can be shared among different threads, allowing concurrent access to sensors and motors in a thread-safe way.

Attempts to re-instantiate class EV3 with the same Channel or AsyncChannel lead to an exception: an instance of type EV3 represents a physical EV3 brick and what determines its identity is the underlying channel, uniquely bound to its peer. However, despite the fact that only one lego main callback can be running at a time, multiple threads can be spawned and access the Api object concurrently, since each EV3 object silently runs a background spooler task sending commands and dispatching replies to the right owner thread.

As far as running subsequent lego main callbacks is concerned, a strict type discipline is enforced by the signature of the EV3.run() method: 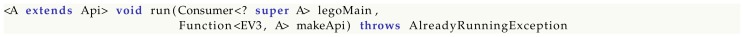


The generic A, constrained to be subtype of EV3.Api, makes this method first-class polymorphic [42], its signature being equivalent to the following polymorphic type:

EV3.run:∀(A⪯EV3.Api)(α⪰A).(α→⌀)∗(EV3→A)→⌀ΔAlreadyRunningException

This type-level design choice allows for reuse of the same EV3 object to run different lego main callbacks picking a different argument of type α, as long as this α is a supertype of A. In a scenario where extra methods are required by the lego main, the type of its argument would have to match A. Admittedly, subtyping rules over functional interface types in Java 8+ would not require the signature of EV3.run() to explicitly support contravariance over the domain type by means of a wildcard in the type argument of the Consumer function [43], although annotating it makes it clearer and open to possible future changes of how the Java type system behaves with respect to lambdas. However, the second argument to the run method, i.e., the construction function, prevents possible wrong reuses by constraining the programmer to explicitly provide a way for instantiating the object of type A.

To further clarify this aspect, we make a few examples. Suppose the following subclasses of EV3.Api exist:

$$\begin{array}{ccc} \mathrm{CustomApi} & ⪯ & \mathrm{EV}3.\mathrm{Api}\\ \mathrm{ExtCustomApi} & ⪯ & \mathrm{CustomApi}\\ \mathrm{AnotherCustomApi} & ⪯ & \mathrm{EV}3.\mathrm{Api} \end{array}$$

In addition, the following *lego main* functions are available:

$$\begin{array}{ccc} \mathrm{legoMain} & : & \mathrm{EV}3.\mathrm{Api}\to ⌀\\ \mathrm{customLegoMain} & : & \mathrm{CustomApi}\to ⌀\\ \mathrm{extCustomLegoMain} & : & \mathrm{ExtCustomApi}\to ⌀\\ \mathrm{anotherCustomLegoMain} & : & \mathrm{AnotherCustomApi}\to ⌀ \end{array}$$

Then, among the following reuses of the same EV3 object with different lego main callbacks, some that are statically accepted by the compiler and others are rejected: 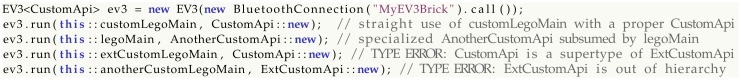


From a programmer’s perspective, a lego main function always accepts subtypes of its argument, not supertypes, in the same way as subsumption applies to arguments in method calls in Java. Finally, it is worth noting that calling ev3.run(this::legoMain) is equivalent to ev3.run(this::legoMain, EV3.Api::new), though the (only) constructor of the EV3.Api class has protected visibility and cannot therefore be referenced from the scope of another class.

### 6.2. Safe Concurrency

In Legodroid, concurrent computations are wrapped by future computations [44], also known as promises [45]. The implementations extend Android FutureTask [46] and the get() method performs lazy evaluation, caching the result for subsequent calls. In class EV3.Api, method execAsync(), belonging to the mid level API, is the basic primitive provided by the library for performing future computations by means of a lambda expression. It executes the callback passed as argument within the default Android single-thread serial executor [47] and returns a FutureTask<T>, i.e., a delayed computation over a value of type T. The type of execAsync() can be formally described with the following polymorphic type scheme [48]:

$$\begin{array}{ccc} \mathrm{exeAsync} & : & \forall \alpha .\;(⌀\to \alpha )\to \mathrm{Future}<\alpha > \end{array}$$

Thanks to Java 8+ lambdas [49,50], wrapping any code block into a closure is syntactically convenient. All methods communicating with the EV3 brick, such as getPercentValue() and getSiValue(), are implemented by calling execAsync() for decoding the data contained in the Reply at the byte level. This mechanism for processing replies to command requests is related to how EV3.SpooledAsyncChannel works: it converts a synchronous Channel into an asynchronous AsyncChannel by spawning an AsyncTask in the background acting as a spooler service, hence the constructor type signature EV3.SpooledAsyncChannel:∀P.Channel<P>→AsyncChannel<P>, where the generic P. The spooler background thread constantly reads from the underlying synchronous channel and dispatches incoming replies to the right *owner* thread, allowing concurrent communication with multiple threads sending commands to the EV3. When a reply is received from the EV3, the spooler signals the relevant FutureReply object that will host the actual reply, waking up its owner thread.

The decoding of replies is performed asynchronously by dozens of short-living threads belonging to the Android thread pool: any input operation, from reading sensors to moving motors, is wrapped within a future computation—in other words, the whole high level API returns objects of type Future<τ>, for some type τ. Users must call the get() of a Future<T> object in order to retrieve the result of type T; subsequent calls do not trigger the computation again, enabling a form of lazy evaluation [51]. Postponing get() calls to the point in code where the result is truly needed may lead to minor performance gains due to massive concurrency, depending on the level of support from the Dalvik virtual machine [52] for fine-grained future computations [53,54].

### 6.3. Improving Sensor Accuracy

LEGO Mindstorms are equipped with a number of sensors: Legodroid performs a basic processing of raw data coming from such sensors, representing values with strong types to aid the programmer in abstracting from the firmware level. The standard sensor set consists of the following items:

**Light** This produces a color value either in RGB format or as an enumeration value supporting a small set of constant colors (At the time of writing, the RGB mode produces random readings on the EV3 even though it appears to be supported. Future versions of the firmware may fix this behavior and Legodroid would immediately start to return correct readings.).
**Gyroscope** It either outputs the angle value in degrees or the rotational speed in degrees per second.
**Pressure** This consists of a button that simply maps onto a boolean value.
**Ultrasound** By emitting ultrasonic waves, this sensor measures the distance from solid obstacles either in centimeters or inches.

**Figure 7:**
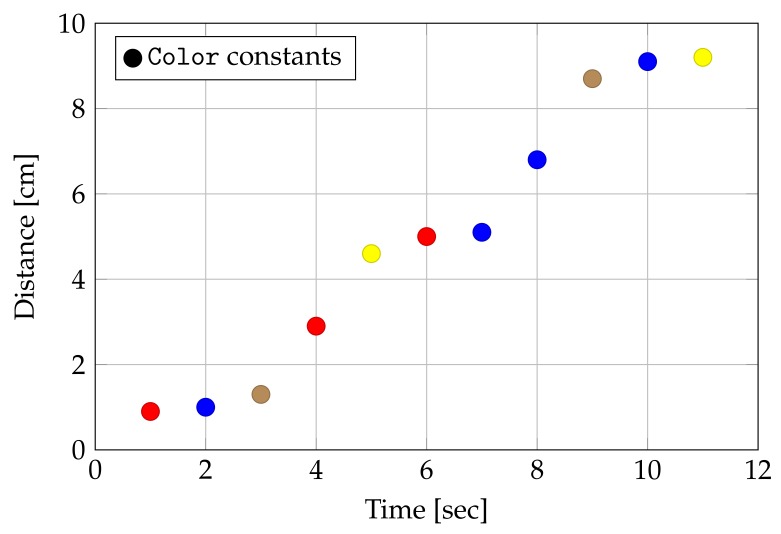
Light sensor reading of a brown object.

Unfortunately, the light sensor happens to be quite imprecise: multiple readings performed over time often produce unstable color values, making the sensor quite hard to use proficiently in virtually any application. Figure 7 shows how a light sensor reads unreliable colors once positioned in front of an object—namely, a brown table under good artificial illumination. The robot is eventually moved and re-positioned at different distances for three seconds; movements take approximately 1 s, as it can been seen from the graph in Figure 7.

Programmers can fix such inaccuracy implementing an algorithm for averaging out readings over time, designing it as subclass of the Api class. The type-driven approach helps in implementing such algorithms in a robust way. As discussed in Section 6.1, the library explicitly supports extensions to the EV3.Api class enforcing a design discipline by abstracting things at two levels:

- at the sensor level, where each class representing a sensor (LightSensor in our case) can be customized and the relevant factory method overridden within the Api class;
- at the data-retrieval level, where each customized sensor subclass (AveragingLightSensor extends LightSensor in our case) can either add extra methods or override the behavior of the existing ones.

In the following sample code, we extend the default EV3.Api class by specializing the factory method responsible for creating instances of the light sensor.

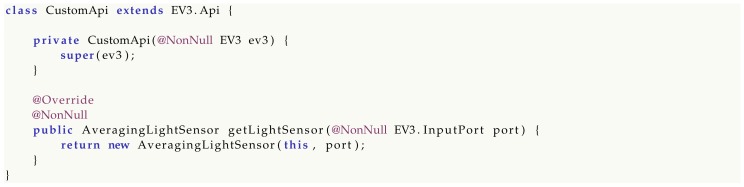



Java supports co-variant return type when overriding methods [55], allowing client code to exploit the additional functionalities provided by the subclass, if needed. Class AveragingLightSensor below extends the LightSensor class provided by the library by overriding the getColor() method.

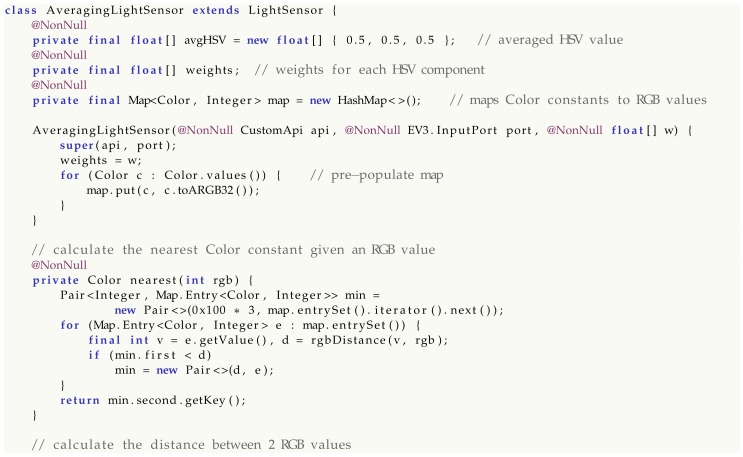

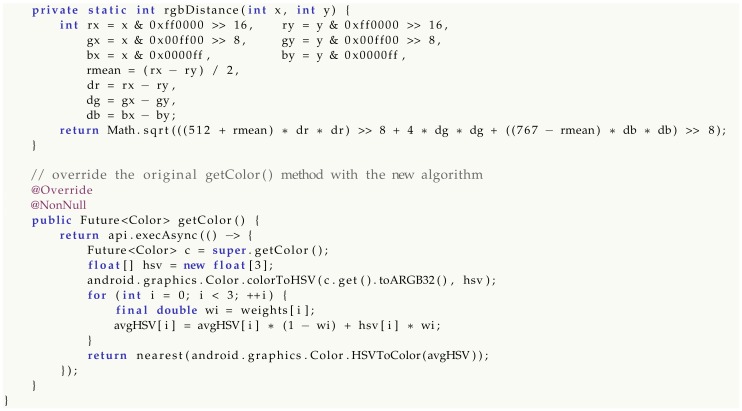





Overriding getColor() replaces the unwanted default behavior in a transparent way for client code. What is interesting is how the execAsync() method—belonging to the mid level API described in Section 4.1 and discussed in detail in Section 6.2—allows for a quick re-wrapping of the semantics inherited from the superclass. It is easy to return the required object of type Future<T> from within a lambda that computes the new value of type T through the desired algorithm. Should the value returned by the original method be needed for the computation, as in our case, super invocations can be safely performed within the closure itself.

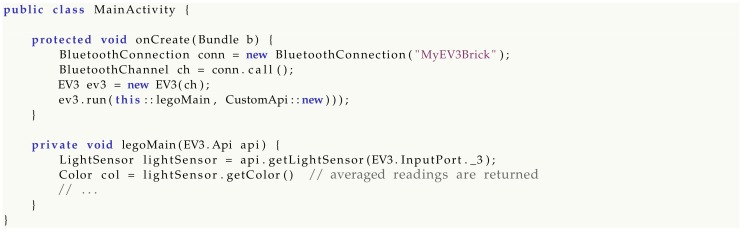



The algorithm is parametric over a triplet ω where, for each integer i∈[0,2], the component ω[i]∈R is a probability within the range [0,1], and, where ω[0] represents the weight of the Hue, ω[1] the Saturation, and ω[2] the Brightness. The algorithm converts the original color from RGB to HSV, which is a preferable color space for this kind of manipulation [56], and performs a simple weighted average of each HSV component using the ω triplet as probability distribution. Calling the algorithm over time tends to produce more stable colors.

The object-oriented version of the algorithm carries on the last averaged output in a field member, meaning each instance of AveragingLightSensor keeps its own value for its entire existence in memory. The procedural version depicted in Algorithm 1 treats such averaged value as both an input and output of the algorithm. Setting up the ω triplet wisely helps in getting quality results: flat $\frac{1}{2}$ probabilities would do the job, but the weight of the Hue should better be different to the weight of the Brightness, due to how the human retina perceive light through two different detectors (cones and rods). Lower probability means that the input component has less weight than its averaged counterpart—an example of a fine-tuned triplet is ω=(0.2,0.6,0.4), where the Hue is more resilient to changes than brightness and saturation.
**Algorithm 1:** Reconstructing inaccurate colors collected by the light sensor (Procedural Version).
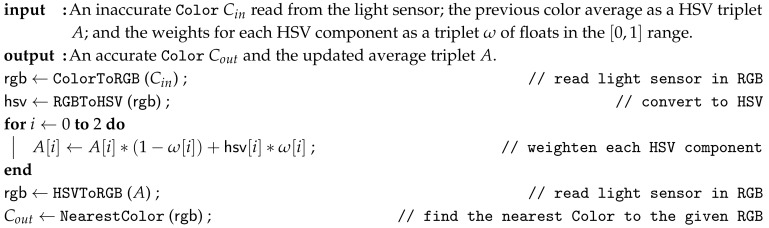


In the last line of the algorithm, to find the nearest color means to convert the averaged color from HSV back to RGB and pick the color constant whose RGB value is less distant from it. Calculating the distance between two RGB colors is an unfinished quest in literature [57]: the formula suggested by CIE [58] takes into account the human-eye factor but is computationally expensive [59] and considered inaccurate in certain cases [60]. A low-cost approximation formula exists [61] for calculating the weighted euclidean distance ΔC between two RGB colors $C_{1}$ and $C_{2}$:

$$\begin{array}{ccc} \bar{r} & = & \frac{C_{1,R}+C_{2,R}}{2}\\ \Delta R & = & C_{1,R}-C_{2,R}\\ \Delta G & = & C_{1,G}-C_{2,G}\\ \Delta B & = & C_{1,B}-C_{2,B}\\ \Delta C & = & \sqrt{\left(2+\frac{\bar{r}}{256}\right)\cdot \Delta R^{2}+4\cdot \Delta G^{2}+\left(2+\frac{255-\bar{r}}{256}\right)\cdot \Delta B^{2}} \end{array}$$

**Figure 8:**
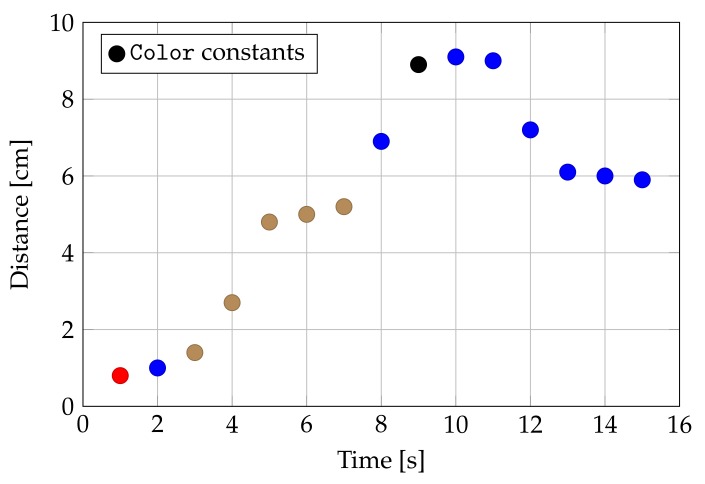
Color reconstruction over time at different distances for two objects: a brown object is replaced at the 8th second by a blue one.

The rgbDistance() method in class AveragingLightSensor is an implementation of this. In Figure 8, we show the output of our color averaging algorithm: for the first seven seconds, the same brown object is pointed, positioning at various distances like in Figure 7; at the 8th second the light sensor is pointed to a different blue-colored object. The algorithm needs a couple of readings to produce a stable color after a change, which is an acceptable compromise for real-world applications. Increasing the sample rate would decrease the time required by the algorithm to stabilize.

## 7. Evaluation

### 7.1. In-Depth Impact Evaluation

One of the major claims of Legodroid is that its strict type discipline prevents the programmer from producing bugged code. In this section, we explore the perimeter of such discipline in order to understand what kind of errors are actually detectable at compile time and how far misuses can get to break the safety granted by the library.

Consider a simple Android app accessing the gyroscope sensor and reading its data in a loop: 
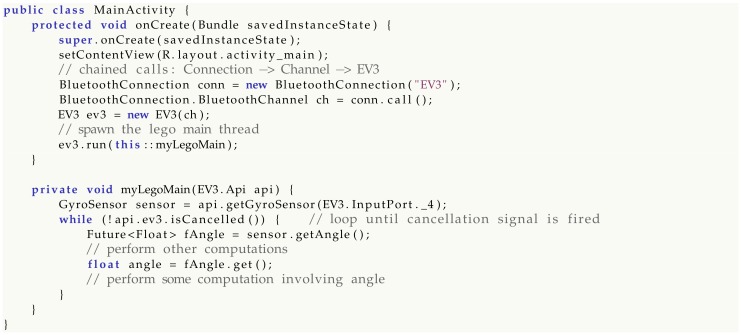


The onCreate() method is the main entry point of an Android app: a precise sequence of calls must be performed here in order to get the lego main run. Any attempt at swapping code lines or at omitting part of the call chain would lead to compile-time errors, giving programmers no room for mistakes in the simplest scenarios.

Android, however, does not always follow a plain serial flow and code is often spread across multiple callbacks. Code decentralization is a common source of bugs due to state invalidity, uninitialized objects and null pointers. Suppose a scenario in which an app wants to read a sensor when some UI event takes place, for example when a button is pressed. Legodroid does not provide any way to access the EV3.Api object from outside the lego main callback, forcing the programmer to exploit closures and related scoping rules [41]. Any attempt at accessing sensors from a different context would not compile.

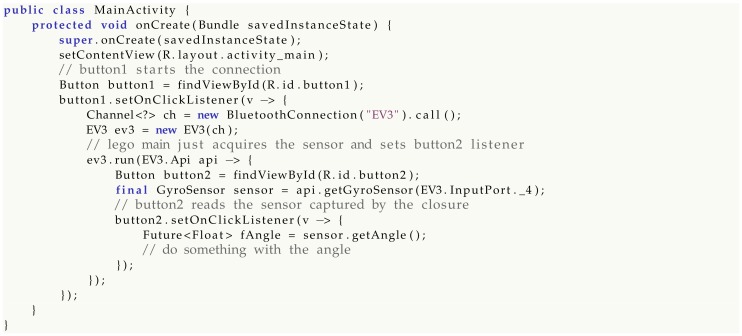



The type-driven patterns imposed by the library can be respected through a wise use of scoping, closures and other language features coming from the functional world. Safety comes at the cost of mastering such programming techniques, in addition to the principles in Section 3.

Many programmers unfortunately employ bad habits when it comes to coding practices: declaring unneeded class fields or binding variables prior to the program point where they are truly needed are common examples. Legodroid cannot prevent this from happening through static checks, therefore silly code can always find a way to break the program, for example by storing the api argument on a field for later use: 
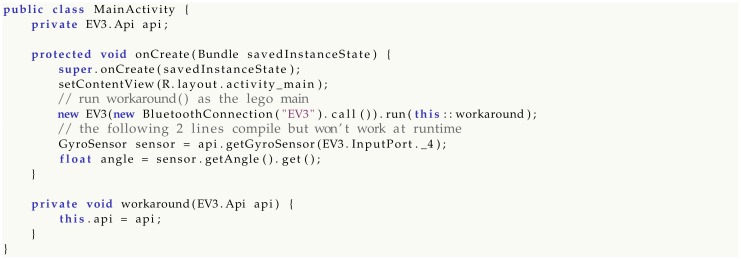


Getting the GyroSensor and reading values would lead to a runtime error due to race conditions occurring on the api field, as the assignment performed by the workaround() method is executed by another thread (see Section 6.2 for details). Programmers need to voluntarily hack the library mechanisms, though, in order to implement such a workaround, rendering this scenario unlikely, albeit possible. Respecting the library coding discipline, with its type-driven patterns, is the main constraint of our proposal.

### 7.2. Usability Evaluation

LEGO Mindstorm devices have already been recognized as valuable educational tools to learn the basics of human–robot interaction [62,63,64]. We extensively tested the Legodroid library with undergraduate students of a Software Engineering university course (Bachelor degree in Computer Science, years 2018–2019 and 2019–2020, DAIS, Università Ca’ Foscari Venezia.). In over two years, more than 200 students split into teams of 3 to 5 people designed and deleloped over 30 apps based on Legodroid performing complex interactions with LEGO Mindstorms devices. Such physical systems ranged across varying genres of systems, among which:

- wheeled machines capable of processing the environment via sensors (both in the robot and in the Android device) in order to collect objects and avoid obstacles;
- an ink-jet printer capable of rendering an input image acquired by the smartphone camera, by moving a pen up and down on a scrolling paper;
- an mp3 player with a physical user interface: by moving levers and pressing buttons, the Android device plays/pauses a song, skips to the next track, raises the volume, etc.;
- a motorized crane capable of carrying and moving objects;
- a weight scale for small objects to explain the theoretical definition of weight as f=ma: makes use of the pressure sensor to detect when the equation is balanced while giving power to the motors lifting the object;
- a multiple safe deposit box, whose chambers can be accessed by means of a password on the companion mobile app, which activates motors for turning the safe interior and open the right door;
- a color Sudoku interactive solver;
- a pills dispatcher, splitting apart a bunch of pills on a color basis in different boxes, and delivering them according to a given schedule.

**Figure 9:**
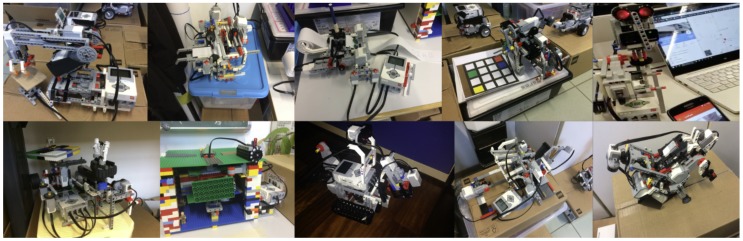
A selection of LEGO Mindstorms robots featured by Ca’ Foscari CS bachelor students.

Figure 9 shows a selection of such LEGO devices. Apps were supposed to exploit the additional computational power of Android, interacting with the EV3 in a non-trivial way: most of the apps use sensors to detect the presence of objects or the distance to them, they process such information in different ways, and eventually they activate the motion of physical parts of the LEGO system.

The Android side allows for sophisticated companion apps, with rich UIs getting and showing relevant information, and allows the user to control the robot.

We observed that all teams welcomed the type-driven patterns offered by the library. Not all students admittedly grasped the most advanced coding practices, which is reasonable for junior developers, though this did not prevent them to take advantage of such patterns. This sense of guidance and safety has been particularly appreciated by all teams, arguably counterbalancing the initial effort to become familiar with the programming patterns.

The adoption of the Legodroid library allowed students to focus on designing smart algorithms and advanced interaction methods rather than getting lost in technical problems related to communication, concurrency, and synchronization issues. The robustness of the resulting systems dramatically improved with respect to the projects developed in the previous editions of the same experiment: in the past years (Bachelor degree in Computer Science, academic years 2016–2017 and 2017–2018, at DAIS, Università Ca’ Foscari Venezia.), teams made use of leJOS for developing programs natively running on the EV3. Despite a simpler setting and the absence of interoperability, teams using leJOS had longer development cycles and mostly deployed code plagued by bugs and weird behaviors at different levels; in some cases, they were not even able to finalize the project in due time.

After using Legodroid, teams reported their appreciation for immutable data types, allowing safe reconstruction of data structures in a stateless fashion that fits how Android transits through different components. Programming with futures instead of event-driven callbacks has been highly appreciated as well.

### 7.3. Limitations and Future Work

Legodroid allows developers to access any sensor or actuator connected to the EV3, though, in order to exploit the capabilities of the Android device, applications have to mix code dealing with Legodroid with code dealing with the Android SDK for accessing sensors on the smartphone.

For example, custom code is needed for exploiting the gyroscope sensor equipped on the smartphone and integrating the incoming data with the data coming from the original LEGO Mindstorms gyrosensor. Future updates of the library may provide a generic mechanism for implementing customizable *virtual sensors*. A programmer may want to implement a virtual sensor that merges data coming from two different physical sensors in a transparent way to the application code. Fixing inaccurate data coming from a sensor by interpolating it with data coming from another sorce is desirable in many real-world scenarios, especially when the smartphone could be physically installed onto the LEGO robot as an additional device attached to it. The Legodroid API may support these scenarios by providing an abstract Sensor class and allowing developers to define a custom virtual sensor by means of a new subclass. The inherited primitive for data acquisition (i.e., a get() method) can then be overridden with a custom behavior.

## 8. Conclusions

The Legodroid library presented in this work offers an effective tool to acquire the basic skills to design systems in which the user interacts with robots equipped with sensors. The same approach, based on rigorous use of types and on the adoption of precise design patterns, deserves to be adopted also in other scenarios, not only for educational purposes but also for industrial development. In particular, the aim of our current work is to bring high level programming methodologies and tools to PLC software development. In a field traditionally bound to low-level coding practices, we argue that raising the quality of the development process is a relevant challenge and that introducing a strong discipline over types and other robust programming patterns and habits may result also in more innovative human–robot interaction solutions.
